# Spotlight on the Granules (Grainyhead-Like Proteins) – From an Evolutionary Conserved Controller of Epithelial Trait to Pioneering the Chromatin Landscape

**DOI:** 10.3389/fmolb.2020.00213

**Published:** 2020-08-21

**Authors:** Vignesh Sundararajan, Qing You Pang, Mahesh Choolani, Ruby Yun-Ju Huang

**Affiliations:** ^1^Center for Translational Medicine, Cancer Science Institute of Singapore, National University of Singapore, Singapore, Singapore; ^2^Department of Obstetrics and Gynaecology, National University of Singapore, Singapore, Singapore; ^3^School of Medicine, College of Medicine, National Taiwan University, Taipei, Taiwan; ^4^Graduate Institute of Oncology, College of Medicine, National Taiwan University, Taipei, Taiwan

**Keywords:** Grainyhead, Grh, Grainyhead-like 2, GRHL2, epithelial-to-mesenchymal transition, pioneer factor, epithelial differentiation, epigenetics

## Abstract

Among the transcription factors that are conserved across phylogeny, the *grainyhead* family holds vital roles in driving the epithelial cell fate. In *Drosophila*, the function of *grainyhead* (*grh*) gene is essential during developmental processes such as epithelial differentiation, tracheal tube formation, maintenance of wing and hair polarity, and epidermal barrier wound repair. Three main mammalian orthologs of *grh*: Grainyhead-like 1-3 (GRHL1, GRHL2, and GRHL3) are highly conserved in terms of their gene structures and functions. GRHL proteins are essentially associated with the development and maintenance of the epithelial phenotype across diverse physiological conditions such as epidermal differentiation and craniofacial development as well as pathological functions including hearing impairment and neural tube defects. More importantly, through direct chromatin binding and induction of epigenetic alterations, GRHL factors function as potent suppressors of oncogenic cellular dedifferentiation program – epithelial-mesenchymal transition and its associated tumor-promoting phenotypes such as tumor cell migration and invasion. On the contrary, GRHL factors also induce pro-tumorigenic effects such as increased migration and anchorage-independent growth in certain tumor types. Furthermore, investigations focusing on the epithelial-specific activation of *grh* and GRHL factors have revealed that these factors potentially act as a pioneer factor in establishing a cell-type/cell-state specific accessible chromatin landscape that is exclusive for epithelial gene transcription. In this review, we highlight the essential roles of *grh* and GRHL factors during embryogenesis and pathogenesis, with a special focus on its emerging pioneering function.

## Introduction

The *grainyhead* (*grh*) transcription factor is a member of the ancestral LSF/Grainyhead gene family. Originally identified in *Drosophila* (previously known as Elf-1 or NTF-1) as an embryonic lethal locus, *grh* mutant *Drosophila* embryos show immature cuticle development, patchy tracheal network and the most notable ‘granular’ head skeleton aberration (Nüsslein-Volhard et al., 1984; Bray and Kafatos, 1991). The LSF/Grainyhead family of proteins are functionally distinctive by their nature of oligomerization and mechanism of DNA-binding module on cognate regulatory sites (Traylor-Knowles et al., 2010). Therefore, the gene family is subdivided into two branches: the LSF/CP2 subfamily and the Grainyhead subfamily, resulting from a major gene duplication event dated more than 700 million years ago (Wilanowski et al., 2002; Venkatesan et al., 2003). These transcription factors bind to *cis*-regulatory elements and control the expression of crucial genes during early embryonic development and tissue homeostasis. The LSF/CP2 subfamily has three mammalian orthologs and evolved from the ancestral gene *gemini* (*dCP2*) in *Drosophila*. Recent critical reviews have discussed the role of LSF/CP2 subfamily members (TFCP2, TFCP2L1, and UBP1) in various aspects of development and human diseases including cancer (Kotarba et al., 2018; Taracha et al., 2018). In this review, we elaborate on the essential functions of *grainyhead* in *Drosophila* and the three mammalian orthologs: Grainyhead-like proteins (GRHL1, GRHL2, and GRHL3). Additionally, we focus on human GRHL2 as an important determinant of the epithelial phenotype during development and as a gatekeeper of epithelial differentiation in several human cancers. Finally, we also discuss in detail the novel pioneering role of *grainyhead* and Grainyhead-like proteins in contouring the chromatin landscape during embryonic development and cancer progression.

The single *grh* gene in *Drosophila* and *Caenorhabditis elegans* exists as multiple orthologs in the vertebrates, which are denoted as *grainyhead-like* (*grhl*) genes. These genes remain evolutionarily conserved from insects to humans. In addition, a varying number of splice variants generated from alternative splicing events and alternative transcriptional initiation sites further highlight the underlying complexity and their gene regulatory networks operating during development and disease progression (Uv et al., 1997; Miles et al., 2017). Of note, three orthologs of *grainyhead* exist in humans: Grainyhead-like 1 (GRHL1), Grainyhead-like 2 (GRHL2) and Grainyhead-like 3 (GRHL3). Each protein contains three annotated functional domains: a transcriptional activation domain (TAD) at the N-terminus; a central DNA-binding domain (DBD) structurally similar to the equivalent of p53, and a dimerization domain (DD) at the C-terminus with unique ubiquitin-like folds. The TAD is the least conserved domain between *Drosophila* and mammalian orthologs, which could be partially due to the presence of an isoleucine-rich segment that has poor conservation across phylogeny (Attardi et al., 1993; Werth et al., 2010). With respect to the human GRHL2 amino acid sequence homology, the DBD holds higher level of sequence identity in all GRHL proteins, when compared to all other domains across the model organisms (Table 1).

**TABLE 1:** Members of the grainyhead/Grainyhead-like transcription factor family and protein sequence homology in nematode, fruit fly, zebrafish, and mammals.

Species	Gene name (Official name)	Aliases	Gene location	Assembly	Splice variants	Amino acid sequence homology (% identity to human GRHL2)			
						**Full length**	**TAD**	**DBD**	**DD**
Round worm (*Caenorhabditis elegans*)	*CP2 domain-containing protein (grh1)*	–	Chr I: 1,259,374-1,268,155	WBcel235: BX284601.5	3	40.5	< 30	54.7	36.1
Fruit Fly (*Drosophila melanogaster*)	*grainy head (grh)*	Dmel\CG42311; DREB; EG:191D12.1; Elf-1; Grh; Ntf; Ntf1	Chr 2R: 17,801,132-17,842,820	BDGP6.28: AE013599.5	8	52.1	< 30	52.1	35.5
Zebrafish (*Danio rerio*)	*grainyhead-like 1 (grhl1)*	fc49a04; wu:fc49a04	Chr 17: 32,391,056-32,426,413	GRCz11: CM002901.2	7	56.9	63.3	73.4	68.2
	*grainyhead-like 2a (grhl2a)*	zgc:110324	Chr 16: 9,807,263-9,830,451	GRCz11: CM002900.2	1	61	63.7	78.9	59.8
	*grainyhead-like 2b (grhl2b)*	grhl2, si:dkey-21k18.2	Chr 19: 12,234,975-12,291,981	GRCz11: CM002903.2	2	70.5	59	86.1	70.7
	*grainyhead-like 3 (grhl3)*	cb467, sb:cb467, si:dkey-221l4.7, wu:fa01c12, wu:fb74c01	Chr 17: 26,965,351-26,977,183	GRCz11: CM002901.2	4	50.3	< 30	55.3	51.8
Mouse (*Mus musculus*)	*grainyhead-like 1 (Grhl1)*	LBP-32, Tcfcp2l2, p61 MGR, p70 MGR	Chr 12: 24,572,283-24,617,391	GRCm38:CM001005.2	4	56.7	59.1	73.4	59.2
	*grainyhead-like 2 (Grhl2)*	0610015A08Rik, BOM, Tcfcp2l3, clft3	Chr 15: 37,233,036-37,363,569	GRCm38:CM001008.2	6	94.7	96.2	98.7	93.2
	*grainyhead-like 3 (Grhl3)*	Get1, Som, ct	Chr 4: 135,541,888-135,573,630	GRCm38:CM000997.2	1	45.6	54.5	57.8	60
Human (*Homo sapiens*)	*grainyhead-like 1 (GRHL1)*	LBP-32, MGR, TFCP2L2	Chr 2: 9,951,693-10,002,277	GRCh38:CM000664.2	8	56.4	59.1	72.6	57.6
	*grainyhead-like 2 (GRHL2)*	BOM, DFNA28, FLJ13782, TFCP2L3	Chr 8: 101,492,439-101,669,726	GRCh38:CM000670.2	6	–	–	–	–
	*grainyhead-like 3 (GRHL3)*	SOM, TFCP2L4	Chr 1: 24,319,322-24,364,482	GRCh38:CM000663.2	9	45.7	50.5	57	64.1

Members of the grh/GRHL family share a similar palindromic DNA-binding motif (AACCGGTT), with different levels of variability for some target genes (Table 2). In *Drosophila*, Grh recognizes DNA regulatory sequences upstream of genes *Ddc* (TG**AACCGGTC**CTGCGG) and *en* (GTGAGCCGGCGA**AACCGGTT**), whereas the binding motif on *Ubx* and *ftz* promoters is (T/C)N**AAC(C/T)GGT(T/C**) (Bray et al., 1988; Soeller et al., 1988; Dynlacht et al., 1989; Wilanowski et al., 2002; Venkatesan et al., 2003). In mammals, GRHL binding motifs display as two adjacent repeats of Grainyhead consensus sequences, with two tandem core CNNG motifs set apart by five bases. For example, the mouse Grhl2 binding site in intron 2 of *Cdh1* (A**AACCAGTC**A**AACCAGT**T) and the promoter of *Cldn4* (A**ATCCAGAG**A**AACTGGTC**) are strikingly similar to the human GRHL2 binding motif on the intron 2 of the *CDH1* (GCA**AACCAGCC**A**AACCAGTT**T) and the promoter of *CLDN4* (GGA**ATCCAGAG**A**AACTGGTC**AG) (Werth et al., 2010; Chung et al., 2016). The invariant CNNG tandem motifs share similarity with the binding motif of Tfcp2l1 from the CP2 family suggesting a close phylogenetic relationship with members of the p53 family (two CNNG set apart by six bases) based on protein folding and the binding of DNA (Kokoszynska et al., 2008). A recent study on the crystal structure of Grhl1/2 DBDs shows that these domains share a common fold with p53, substantiating earlier computational predictions (Ming et al., 2018).

**TABLE 2:** A non-exhaustive list of transcriptional targets of Grh/GRHL factors in literature.

Target gene	Binding region	Species/Model	Regulated by GRHL	Activation/Repression	Function	References
Dopa decarboxylase *(Ddc)*	Promoter/Enhancer	*Drosophila*	Grainyhead	Activation	Hardening of larval and adult cuticles	Bray and Kafatos, 1991; Venkatesan et al., 2003; Mace et al., 2005
Ultrabithorax (*Ubx*)	Promoter	*Drosophila*	Grainyhead	Activation	Differentiation of ‘head skeleton’	Bray and Kafatos, 1991
Engrailed (*en*)	Promoter	*Drosophila*	Grainyhead	Activation	Posterior wing compartment identity	Soeller et al., 1988
Zerknüllt (*zen*), Tailless (*tll*), Scute (*sc*), Sex lethal (*Sxl*)	Promoter	*Drosophila*	Grainyhead	Repression	Developmental patterning, sex determination and cellularization	Harrison et al., 2010
Fasciclin 3 (*Fas3*), Coracle (*cora*), Sinuous (*Sinu*)	Intron 1	*Drosophila*	Grainyhead	Activation	Septate junction proteins that forms barrier epithelia	Narasimha et al., 2008
Stitcher (*stit*)	Intron 2	*Drosophila*	Grainyhead	Activation	Epidermal wound healing	Wang et al., 2009
Misshapen (*msn*), Krotzkopf verkehrt (*kkv*), Tyrosine hydroxylase (*ple*)	Enhancer	*Drosophila*	Grainyhead	Activation	Epidermal wound healing	Pearson et al., 2009
Claudin b (*cldnb*), Epithelial cell adhesion molecule (*epcam*)	Enhancer	Zebrafish	Grhl2b	Activation	Otic development and hearing ability	Han et al., 2011
Engrailed 2a (*eng2a*), CDC42 small effector 1 (*cdc42se1*)	Promoter	Zebrafish	Grhl2b	Activation	Midbrain-hindbrain morphogenesis	Dworkin et al., 2012
Engrailed-1 (*EN1*)	Promoter	Human	GRHL1	Activation	Morphogenesis	Wilanowski et al., 2002
Desmoglein 1 (*Dsg1/DSG1*)	Promoter	Human and Mouse	GRHL1	Activation	Desmosome organization	Wilanowski et al., 2008
Albumin (*Alb*), Carbamoylphosphate synthetase I (*1*), Hepatocyte nuclear factor 4α (*Hnf4*α), CCAAT/enhancer binding protein α (*Cebpa*)	Unknown	Mouse	Grhl2	Repression	Inhibition of hepatocytic differentiation	Tanimizu et al., 2013
Claudin 3 (*Cldn3*), Claudin 4 (*Cldn4*), E-cadherin (*Cdh1*)	Promoter – *Cldn4*, Intron 2 – *Cdh1*	Mouse	Grhl2	Activation	Maintenance of breast epithelial cell identity	Werth et al., 2010
miR-122	Promoter	Human	GRHL2	Repression	Ethanol induced liver injury and fibrosis	Satishchandran et al., 2018
miR-200b/-200a/429	Promoter	Human	GRHL2	Activation	Suppression of EMT	Cieply et al., 2012; Chung et al., 2016
Serine peptidase inhibitor, Kunitz type 1 (*SPINT1*)	Promoter	Human	GRHL2	Activation	Salivary gland development	Walentin et al., 2015; Matsushita et al., 2018
Matrix metalloproteinases (MMPs) - multiple	Unknown	Human	GRHL2	Repression	Suppression of invasion phenotype	Chung et al., 2016; Pifer et al., 2016; Xiang et al., 2017
Forkhead box M1B (*FOXM1B*)	Promoter	Human	GRHL2	Activation	Human papillomavirus associated oropharyngeal cancer development	Chen et al., 2018c, 2
Tumor protein p63 (*TP63/p63*)	Promoter	Human	GRHL2	Activation	Maintenance of epithelial phenotype in human keratinocytes	Mehrazarin et al., 2015
Ovo like zinc finger 2 (*Ovol2/OVOL2*)	Promoter	Human and Mouse	GRHL2	Activation	Epithelial barrier function and palatogenesis	Watanabe et al., 2014; Aue et al., 2015; Carpinelli et al., 2020
v-erb-b2 avian erythroblastic leukemia viral oncogene homolog 3 (*Erbb3/ERBB3*)	Promoter	Human and Mouse	GRHL2	Activation	Suppression of EMT	Werner et al., 2013; Chung et al., 2016
member RAS oncogene family (*Rab25/RAB25*)	Promoter	Human and Mouse	GRHL2	Activation	Regulation of epithelial morphogenesis	Senga et al., 2012; Gao et al., 2013
Rho Guanine Nucleotide Exchange Factor 19 (*Arhgef19/ARHGEF19*)	Promoter	Human and Mouse	GRHL2 and GRHL3	Activation	Maintenance of epidermal differentiation	Caddy et al., 2010; Boglev et al., 2011; Gao et al., 2013
miR-21	Promoter	Human and Mouse	GRHL3	Repression	Maintenance of epidermal differentiation	Bhandari et al., 2013
Transglutaminase 1 (*Tgm1*/*TGM1*)	Promoter	Human and Mouse	GRHL3	Activation	Maintenance of epidermal differentiation	Ting et al., 2005; Boglev et al., 2011
Uroplakin 2 (*UpkII*)	Promoter	Mouse	GRHL3	Activation	Urothelial differentiation	Yu et al., 2009

## Grainyhead and GRHL Factors in Development

In metazoans, two major cell types form the basis of organ development: epithelium and mesenchyme. Epithelial cells are generated first during the embryonic development, while mesenchymal cells are derived from the pre-existing epithelial cells through a process called epithelial-to-mesenchymal transition (EMT) (Hay, 1995; Thiery and Sleeman, 2006; Kalluri and Weinberg, 2009). Epithelial cells usually maintain a strict, aligned cellular polarity (apical and basal surfaces) and remain closely connected to adjacent cells through specialized transmembrane structures, such as tight junctions, adherens junctions, and desmosomes. In contrast, due to their lack of stable cell-cell attachment and apical-basal polarity, mesenchymal cells possess higher migratory abilities and interact extensively with the surrounding extracellular matrix. The earliest developmental EMT occurs during gastrulation, where mesenchymal cells are generated from epithelial epiblast cells. The mesenchyme further condenses to form mesoderm (middle layer of the embryo) and endoderm (inner layer of the embryo), which eventually form the vertebral column, bony appendages and connective tissues (Hay, 2005). However, the epithelia is the stable state of cellular organization that forms the epidermis, the primary layer covering the external surface of the body that provides protection against external physical and mechanical stress. The following sections describe the vital roles of Grainyhead and Grainyhead-like proteins in the epidermis and epithelia.

The recurrence of EMT is also observed during the early development of the nervous system to generate neural crest cells. During the embryonic process termed neurulation, epithelial neural plate (neural ectoderm) bends, invaginates and fuses along the dorsal midline to form a cylindrical structure called the neural tube. Subsequent closure of the neural tube in anterior and posterior directions guides formation of the future brain and spinal cord (Wilde et al., 2014). Meanwhile, the dorsal neuroepithelial cells located close to the neural tube lose intercellular connections, undergo EMT, and migrate away to become neural crest cells (Acloque et al., 2009). Further delamination and migration of neural crest cells navigate to populate multiple niches throughout the embryo, ultimately progress toward terminal differentiation derivatives such as ganglia of the peripheral and enteric nervous system, cardiac valves, bone and cartilage of the facial skeleton (Simões-Costa and Bronner, 2013; Muñoz and Trainor, 2015). Members of the Grainyhead-like family provide distinct regional signals during neurulation, neural crest migration, and neural tube closure that are discussed in detail in an upcoming section.

### *Drosophila* Grainyhead During Epidermal Morphogenesis

In *Drosophila*, the formation of protective exoskeleton called cuticle during the early stages of larval development serves many important functions during the adult life including the protection against water loss and the maintenance of structural framework for locomotion. *grh* remains to be essential for the development of the epidermal barrier and the repair of barriers after wounding. The embryonically lethal larval cuticles of *grh* mutants are multilayered and grossly inflated structures, generating the “blimp” phenotype that are functionally weaker when compared to wild-type cuticles (Uv et al., 1997; Ostrowski et al., 2002; Hemphälä et al., 2003). *grh* mutant embryos carrying induced aseptic epidermal wounds fail to restore the expression of Dopa decarboxylase *(Ddc* in the wound border), one of the two key enzymes contributing for the formation and hardening of larval and adult cuticles. This results in the defective phosphorylation of *grh* by ERK which is required for wound-dependent regeneration of the epidermal barrier (Mace et al., 2005; Geiger et al., 2011; Kim and McGinnis, 2011). Other studies focusing on *Drosophila* epidermal wound healing and amnioserosa (defects in dorsal closure) have identified the direct regulation of Grh on major targets including *stit*, *msn*, *cora*, *sinu*, and *fas3* (Narasimha et al., 2008; Pearson et al., 2009; Wang and Samakovlis, 2012). In addition to controlling the epithelial and epidermal morphogenesis in *Drosophila*, Grh directly regulates the expression of key genes (Table 2) that are involved in the tracheal tube formation (Hemphälä et al., 2003), the maturation of central nervous system (Almeida and Bray, 2005; Khandelwal et al., 2017), and the maintenance of polarity in wing and hair (Lee and Adler, 2004).

### GRHL Family in Epithelial Morphogenesis and Development

**FIGURE 1:**
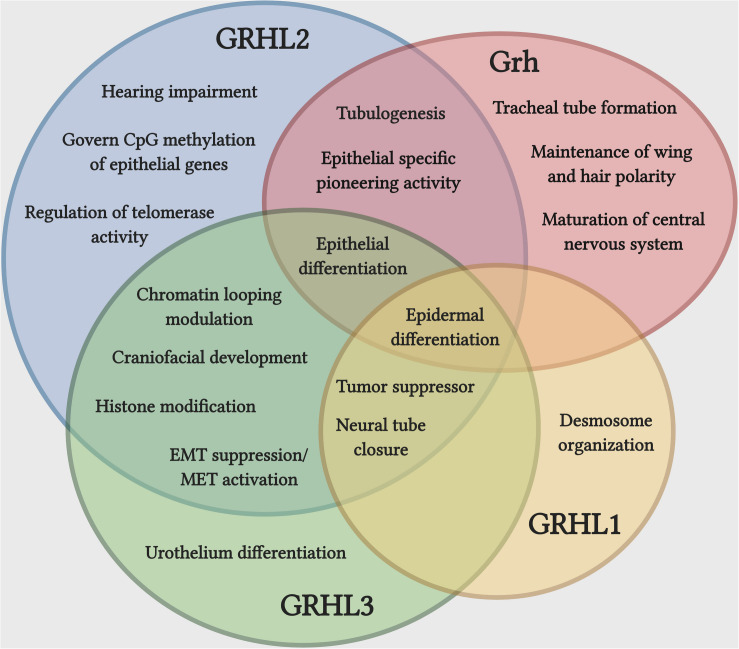
Unique and cooperative functions of Grh and GRHL family during development and disease. Epidermal differentiation is the most common function shared by Grh and GRHL family, while a set of functions associated with epithelial differentiation is carried out by more than one member of the family. The overlapping function does not indicate that they are functionally redundant. Illustration created with Biorender.com.

The GRHL family members play crucial roles during the development of several epithelial tissues (Figure 1). During the development of mouse circumvallate papilla (specialized dome shaped region located at the back of a tongue), knockdown of *Grhl3* significantly alters the epithelial structure and disrupts the epithelial integrity by having high proliferation, low apoptosis, and enhanced migration in epithelial tongues cells of embryonic mice (Adhikari et al., 2017). Several reports have claimed the prominent function of GRHL factors in epidermal integrity. Grhl1 directly controls the expression of the desmosomal cadherin, desmoglein 1 (*Dsg1*) and mice deficient of *Grhl1* show an abnormal desmosome phenotype in the interfollicular epidermis, ultimately delaying the initial skin coat growth and poor hair anchoring to the follicle (Wilanowski et al., 2008).

During early embryogenesis, Grhl2 expression is predominantly observed in several barrier-forming epithelial tissues, including the surface ectoderm, the otic ectoderm, and the gut tube (Auden et al., 2006; Werth et al., 2010). At the molecular level, Grhl2 binds to cis-regulatory elements and controls the timely expression of the apical junctional complex proteins such as the adherens junction component E-cadherin and the tight junction molecules claudin 3, claudin 4 (*Cldn3/4*), and an epithelial-specific member of small guanosine triphosphatase Rab25, which are crucial for epithelial differentiation (Werth et al., 2010; Senga et al., 2012; Tanimizu and Mitaka, 2013). GRHL2 is involved in the epithelial morphogenesis of the lung epithelium and essential for the establishment and maintenance of the epithelial barrier of mucociliary airways. In primary human bronchial epithelial cells, GRHL2 directly or indirectly regulates the expression of proteins that form apical junction assembly and cell polarity (CDH1, TJP1, RAB25) as well as essential proteins that are required to establish barrier function (PVRL4, VAV1, and ESRP1/2). Mutant cells carrying dominant–negative GRHL2 protein consequently fail to form polarized epithelium with barrier function (Gao et al., 2013). Conditional deletion of *Grhl2* in mouse tracheal basal cells and *in vitro* CRISPR/Cas9 genome editing of *GRHL2* in human basal cells have been shown to disrupt the differentiation of ciliated cells via targeting multiple genes in the Notch signaling pathway and ciliogenesis such as *Mcidas*, *Rfx2*, and *Myb* (Gao et al., 2015). In cooperation with Nkx2-1, a homeobox transcription factor, Grhl2 regulates the expression of cell-cell interaction genes such as semaphorins and their receptors, which are crucial for maintaining the lung epithelial identity (Varma et al., 2012). Using mouse lung epithelial cells, the same study shows that Grhl2 binds to the *Nkx2-1* promoter regions and Nkx2-1 binds to the *Grhl2* intronic region and generates a positive feedback loop to reinforce lung epithelial phenotypes. Furthermore, a recent study reports that the loss of *Grhl2* in the developing mice lung epithelium reduces the expression of Elf5, an epithelial-specific transcription factor, and eventually leads to the impaired ciliated cell differentiation and the reduction of distal progenitor cells (Kersbergen et al., 2018).

Grhl3 controls epidermal differentiation and wound-repair by directly regulating the expression of two crucial genes: *Transglutaminase-1*, an enzyme that crosslinks structural components of the superficial epidermis (Ting et al., 2005), and *RhoGEF19*, a RhoA activator of the planar cell polarity (PCP) signaling pathway (Caddy et al., 2010). Grhl3 functionally interacts with the LIM-only protein LMO4 to regulate the differentiation of the epidermis, where mice lacking functional Grhl3 and LMO4 expression show severe defective skin barrier formation and failure of eyelid development affecting the expression of multiple genes linked to the epidermal terminal differentiation and F-actin cable formation (Yu et al., 2006, 2008; Hislop et al., 2008). Mice deleted for epidermal specific *Grhl3* have digit fusion (syndactyly) due to abnormal adhesion of the periderm covering the developing digits (Kashgari et al., 2020). In autosomal-recessive ectodermal dysplasia syndrome, whole exome-sequencing from affected individuals revealed the presence of homozygous mutations in the *GRHL2* locus. These mutant keratinocytes showed changes in the cellular phenotype and failure to form intact cell-cell junctions, partly due to the cytoplasmic translocation of GRHL2 (Petrof et al., 2014). Interestingly, cytoplasmic translocation of GRHL3 induces the activation of the non-canonical Wnt signaling pathway involving PCP genes, and eventually alters the mechanical properties essential for enduring tensile force during epithelial differentiation (Kimura-Yoshida et al., 2018). In additional to the pulmonary and epidermal epithelium, another major tissue that plays an important role in the barrier function is the urothelial membrane, which controls the selective movement of water and solutes between urine and tissues. Grhl3 is highly expressed in mature umbrella cells of the bladder epithelium. It directly targets the expression of *uroplakin II*, a major protein component of the asymmetric urothelial membrane plaques, thereby regulating the terminal differentiation and barrier function of the bladder epithelium (Yu et al., 2009).

### GRHL Family in Neural Development

During neurulation, the single-layered neurepithelium distinguishes into two different cell fates: neural ectoderm and surface (non-neural) ectoderm prior to neural tube closure. The fate choice between neural and surface ectoderm is highly regulated through signaling interplay of Wnt, FGF and BMP activity (Murry and Keller, 2008). At the molecular level, canonical Wnt signaling mediated Grhl3 expression is essential for the specification of surface ectoderm cell fate, whereas, repression of Grhl3 by Dickkopf1 (Dkk1), a canonical Wnt signaling antagonist, leads to the specification of neural ectoderm cell fate (Ting et al., 2003; Kimura-Yoshida et al., 2015). Thus, the balance between Wnt controlled Grhl3 activation or repression regulates the binary cell fate choice of neural and surface ectoderm identity, which is essential for subsequent neural tube closure. The remodeling of five or more polarized epithelial cells converging radially around a central point of fusion to form transient “rosette” like structures are identified during the formation of multiple organ systems, including surface ectodermal lineage specification (Afonso and Henrique, 2006; Nishimura and Takeichi, 2008; Harding et al., 2014). Genetic fate mapping using *Grhl3*^*Cre/+*^ mice have reported that the rosette forming cells of the surface ectoderm are Grhl3-expressing lineage cells, and Grhl3 mutants showed severe disruption of rosette formation, exhibiting fully penetrant spina bifida (Molè et al., 2020; Zhou et al., 2020). Single-cell RNA sequencing of neural rosettes *in vitro*, generated from human induced pluripotent stem cells, showed GRHL2 and GRHL3 were highly expressed in early rosettes highlighting the role for GRHL TFs in neurulation in humans (Shang et al., 2018). Therefore, these findings denote an earlier role for GRHL factors in neurulation, such as lineage specification, prior to its contribution in neural tube closure.

Delamination and migration of neural crest cell from the border of the surface and neural ectoderm involves activation of EMT. Although GRHL factors are potent suppressors of EMT and associated phenotypes, depletion of *grhl3*/*Grhl3* in zebrafish and mouse embryos do not affect any stages of neural crest cell development and activity (Dworkin et al., 2014; Kimura-Yoshida et al., 2015). This might indicate that fate specification of surface ectoderm in the neural plate border is partly driven by GRHL factors. However, the involvement of other GRHL factors in neural crest cell migration remains to be explored.

Following the delineation of the neurepithelium, the developing neural tube converges and fuses along the midline to form complete the neural tube closure event. Among the GRHL family, disruption of GRHL2 and GRHL3 functions generated severe neural tube defects. Loss of Grhl2 expression in the surface ectoderm resulted in abnormal mesenchymal phenotypes, with increase in vimentin expression and downregulation of epithelial genes such as *Fermt1*, *Esrp1*, and *Tmprss2*, eventually resulting in neural tube closure defects (Pyrgaki et al., 2011; Ray and Niswander, 2016). In the Grhl2-null mutants, the expression levels of two Grhl2 direct targets, E-cadherin (*Cdh1*) and Claudin 4 (*Cldn4*), are significantly reduced in the surface ectoderm leading to neural tube defects (Werth et al., 2010). In contrast, overexpression of Grhl2 could also be the underlying cause of defective neural tube closure in Axial defects mutant mouse (Brouns et al., 2011). Similarly, Grhl3-null mutants exhibited fully penetrant spina bifida, and lack of Grhl3 expression in the hindgut caused curly tail phenotype, which occurs during the final stages of neural tube closure (Ting et al., 2003; Auden et al., 2006; Gustavsson et al., 2007). Moreover, Grhl2 and Grhl3 exhibit cooperative activity during neurulation closure at the forebrain/midbrain boundary and spinal closure from mid to lower thoracic region (Rifat et al., 2010). Taken together, members of the GRHL family play critical roles during several stages of neural development and dysregulation of these factors during neural development renders severe impact on neural tube closure.

## GRHL in Pathophysiology

An array of studies has reported the implication of GRHL members in multiple human diseases including cancers. Essential functioning of GRHL factors are implicated during carcinogenesis as well as during tumor suppression indicating that these factors play complex and controversial roles in regulating different cancer entities. In the following section we highlight several prominent findings that describe the essential roles of GRHL factors in pathophysiology.

### Tumor Promoting Roles of GRHL Members

Few recent studies have demonstrated the association of expression levels of GRHL members with patient outcomes during cancer progression. In colorectal cancer, higher expression levels of GRHL1 and GRHL3 are associated with worse disease-free survival, whereas low levels of all three members confer better overall survival of patients (Yuan et al., 2020). GRHL2 expression is enriched in human breast cancer stem cell-like subpopulation and is included in a 31-gene signature predictive of distant metastasis in estrogen receptor–negative breast cancer cohorts (Leth-Larsen et al., 2012). Gene correlation analysis of a breast cancer cohort showed that higher expression of GRHL2 is correlated with worse relapse-free survival in Luminal A, Luminal B, HER2^+^, and Basal-like subtypes (Mooney et al., 2017). Furthermore, overexpression of GRHL2 in breast cancer cell lines showed significant increase in migration, invasion potential and also correlated with unfavorable breast cancer patient characteristics – grade III tumors and large tumor size at the time of diagnosis (Yang et al., 2013). High expression of GRHL2 is also observed in pancreatic cancer patients with worsened overall survival (Wang et al., 2019).

At the molecular level, GRHL2 directly regulates the expression of the EGFR family member *ERBB3* and the Wnt ligand *Wnt7A*, and overexpression of GRHL2 in metastatic breast cancer cells exhibit increased anchorage-independent growth, migratory and invasive potential (Xiang et al., 2012; Werner et al., 2013). Aberrant activation of ERBB3 potentially forms heterodimers with ERBB2 that directly contributes to decreased survival rate coupled with increased resistance to chemotherapy (Liu et al., 2007, 3; Sithanandam and Anderson, 2008; Garrett et al., 2011). Conditional deletion of *Grhl2* prevents oral cancer development in a chronic, chemically induced carcinogen model, when compared to the aggressive tumor formation in *Grhl2* wild-type mice (Chen et al., 2018b). In addition, the study also identifies that GRHL2 mediated activation of MAP kinase signaling and repression of TGF-β signaling in oral squamous cell carcinoma cell lines, dually render tumor promoting effects during the early stages of carcinogenesis. The role of GRHL2 in prostate cancer seems to be mainly oncogenic, as its expression is higher in prostate cancer tissue samples (Danila et al., 2014; Paltoglou et al., 2017). *Paltoglou et al*. showed that the loss of GRHL2 via silencing resulted in the loss of androgen receptor (AR) expression and demonstrated the presence of a positive feedback loop between GRHL2 and AR to promote prostate cancer growth (Paltoglou et al., 2017). In addition to driving AR expression, GRHL2 also acts as an AR transcriptional co-activator that enhances the oncogenic AR signaling pathway in prostate cancer progression. Furthermore, the oncogenic role of GRHL2 is observed in hepatocellular carcinoma (Tanaka et al., 2008), esophageal cancer (Shao et al., 2017), oral squamous cell carcinoma (Kang et al., 2009; Chen et al., 2016), and colorectal carcinoma (Quan et al., 2014, 2015).

### GRHL Members as Tumor Suppressors

In squamous cell carcinoma, GRHL3 functions as a strong tumor suppressor, where the deletion of *Grhl3* in keratinocytes leads to hyper-proliferation epidermal keratinocytes that are more prone to chemical carcinogen induced spontaneous squamous cell carcinoma formation (Darido et al., 2011). Mechanistically, *Grhl3* depletion in keratinocytes leads to the loss of tumor suppressor *Pten* expression, which induces the activation of PI3K/AKT/mTOR signaling and the oncogenic miR-21 expression, culminating in the formation of aggressive and poorly differentiated squamous cell carcinoma. Oncogenic Ras-mediated *Grhl3*^–/–^ mouse epidermal keratinocytes were more prone to tumorigenesis by upregulating the miR-21 levels (Bhandari et al., 2013). Similarly, in a two-stage chemical skin carcinogenesis model, over 40% of benign papilloma developed into squamous cell carcinoma in *Grhl1*^–/–^ mice, when compared to one-fourth of such tumor formation in *Grhl1*^+/+^ mice, due to the severe impairment of epidermal barrier and aberrant terminal differentiation of keratinocytes (Mlacki et al., 2014). In neuroblastoma, patients with high levels of GRHL1 expression show favorable prognosis, consistent with the suppressed tumor growth phenotype seen in the xenografts carrying forced GRHL1 expression in *MYCN*-amplified neuroblastoma cells (Fabian et al., 2014). At the molecular level, co-recruitment of MYCN and HDAC3 to the *GRHL1* promoter represses its transcription. Esophageal squamous cell carcinoma with low expression of GRHL1 levels show poor differentiation and the patients are associated with reduced overall survival rate (Li M. et al., 2019). The tumor suppressive role of GRHL2 is largely mediated though the suppression of EMT which is summarized in the following section.

### GRHL Members as a Determinant for EMT and MET Execution During Cancer Progression

EMT and MET (mesenchymal to epithelial transition, the reversal of EMT) functionally dictate the cellular dedifferentiation and differentiation status respectively and determine the nature of cellular behavior. Although EMT/MET processes occur spontaneously during fundamental events such as gastrulation, neural crest dissemination and organogenesis, execution of EMT/MET is also observed during wound healing, fibrosis and cancer (Thiery and Sleeman, 2006). Overcoming years of speculations about the reactivation of reversible EMT and MET program during cancer progression, compelling evidences from *in vitro*, *in vivo* and clinical findings support the crucial roles of EMT and MET during cancer progression (McInnes et al., 2015; Santamaria et al., 2017; Francart et al., 2018; Rios et al., 2019; Williams et al., 2019). In brief, acquisition of mesenchymal trait through EMT is regarded as an essential feature for epithelial-derived cancer cells to successfully metastasize the surrounding tissues and distant organs. Being a pivotal gatekeeper of epithelial integrity, GRHL2 suppresses EMT in a multipronged manner across cancer entities. Firstly, multiple independent investigations have shown that GRHL2 controls the ZEB1/miR-200 regulatory axis. The EMT inducer ZEB1 and epithelial-phenotype reinforcing microRNA-200 (miR-200) family members reciprocally control the expression of each other generating a double-negative feedback loop (Brabletz and Brabletz, 2010). The expression of ZEB1 drives the cancer cells to undergo EMT, whereas restoration of miR-200 expression is vital for cells to undergo epithelial differentiation or MET. On one hand, GRHL2 directly suppresses the expression of the EMT inducer ZEB1 in breast (Cieply et al., 2012; Werner et al., 2013), ovarian (Chung et al., 2016), bladder cancers (Shen et al., 2020), and sarcoma (Somarelli et al., 2016). On the other hand, GRHL2 activates the expression of miR-200 family members through direct promoter binding in oral (Chen et al., 2016), ovarian (Chung et al., 2016) cancers and sarcoma (Somarelli et al., 2016).

Secondly, GRHL2 suppresses TGF-β mediated migratory and invasive capabilities of gastric (Xiang et al., 2017), breast (Cieply et al., 2012; Werner et al., 2013), and oral (Chen et al., 2018b) cancer cells, where the activation of TGF-β signaling cascade is a significant inducer of EMT in tumor progression (Heldin et al., 2012). Thirdly, re-expression of GRHL2 in mesenchymal-like cells induces MET effects by restoring the expression of epithelial components such as E-cadherin, ZO-1 and downregulating mesenchymal markers including Vimentin, Snail, Slug and ZEB1 (Chung et al., 2016; Yang et al., 2019; Shen et al., 2020). Fourthly, GRHL2 expression suppresses stemness properties in CD44^*high*^/CD24^*low*^ mesenchymal subpopulation cells of breast cancer cells and restores the anoikis sensitivity by altering intracellular H_2_O_2_ ROS levels (Cieply et al., 2012; Farris et al., 2016).

EMT-TFs Snail and ZEB1 are known to recruit epigenetic remodelers such as the DNA methyltransferases (DNMTs) and/or polycomb repressive complex 2 (PRC2) to generate a repressive chromatin around epithelial genes (Dong et al., 2012; Fukagawa et al., 2015). GRHL2 also interacts with multiple epigenetic regulators to dually suppress EMT and to induce MET phenotypes. GRHL2 significantly inhibits the histone acetyltransferase coactivator p300 and its activity on mesenchymal genes, which interfered with the branching morphogenesis and EMT of Madin–Darby canine kidney (MDCK) cells (Pifer et al., 2016). In addition, GRHL2 interaction with the histone methyltransferases KMT2C and KMT2D induces MET and ICAM-1 expression in cancer cells, which sensitizes these cells for optimal natural killer (NK) cells mediated activation and target cell killing, suggesting a potential link between the epithelial phenotype and cellular susceptibility to NK killing (MacFawn et al., 2019). Furthermore, using a set of ovarian cancer cell lines, we have shown that during the reactivation of epithelial genes, the presence of GRHL2 is essential for the modification of the epigenetic landscape into a permissive chromatin to allow the transcription of key epithelial genes such as E-cadherin, ESRP1 and OVOL2 (Chung et al., 2019).

Evidences have shown that GRHL members are the gatekeepers of early phenotype transition. Our assessment on a heterogeneous ovarian cancer cell line panel revealed the presence of intermediate cellular phenotypes that dually expressed epithelial and mesenchymal markers (Huang et al., 2013). Consequently, the concept of an ‘EMT spectrum’ has emerged, whereby EMT is regarded as a continuum consisting of multiple, transient intermediate phenotypes collectively referred as the EMT spectrum (Nieto et al., 2016; Yang et al., 2020). The prevalence of multiple intermediate EMT states is observed in breast (Grosse-Wilde et al., 2015; Yamashita et al., 2018), prostate (Ruscetti et al., 2015), and non-small lung cancer (Fustaino et al., 2017). GRHL2 knockdown in ovarian cancer cell lines harboring the epithelial phenotype results in a specific shift of subcellular E-cadherin localization with unaltered total E-cadherin protein abundance, generating a partial EMT phenotype (Chung et al., 2016). In lung cancer cells, GRHL2, OVOL2 and miR-145 play crucial roles in stabilizing the intermediate EMT phenotype (hybrid E/M), while transient knockdown of GRHL2 in cells with hybrid E/M phenotype switches to a complete EMT as evidenced by the disruption of partial EMT specific collective cell migration phenotype to a single cell migration phenotype (Jolly et al., 2016). Moreover, through computational modeling, the same study has predicted that GRHL2 promotes the association of hybrid E/M phenotype with high-tumor initiating stem-like traits, which might be helpful in stratifying patients with higher metastatic risk. Using a genetically engineered mouse model of squamous cell carcinoma, another recent study has identified spontaneous EMT and multiple intermediate EMT subpopulations with characteristic cell surface marker expressions (Pastushenko et al., 2018). Assay for transposase-accessible chromatin using sequencing (ATAC-seq) of these EMT subpopulations revealed the specific enrichment of a GRHL1 motif on the differentially expressed genes especially during the epithelial to early hybrid EMT state but not observed in the late hybrid EMT state or in mesenchymal subpopulations. Although tumor cells dynamically switch between epithelial, mesenchymal and intermediate E/M phenotypes through EMT and MET processes, mechanisms underlying the reversibility or irreversibility of such events are slowly emerging. In an inducible mammary EMT system (HMLE-Twist1-ER), epithelial clonal population (high GRHL2 expression) were susceptible to acquire a hybrid E/M phenotype and showed transient, reversible changes in chromatin accessibility when compared to the mesenchymal clonal population (low GRHL1/2 expression) (Eichelberger et al., 2020). In particular, ATAC-seq of the mesenchymal subpopulation revealed that the specific loss of chromatin accessibility along GRHL1/2 motifs governing loci of epithelial genes is crucial for these cells to enter an irreversible mesenchymal cell state or to resist trans-differentiation. Similarly, via mathematical modeling, another independent study has proposed two mechanisms that drive epithelial cells to resist undergoing EMT or enabling irreversible MET: (i) GRHL2 mediated epigenetic feedback on inhibition of ZEB1 and (ii) stochastic partitioning of biomolecules during cell division to generate different phenotypic subpopulations in regards to EMT (Jia et al., 2020). Altogether, these studies have unraveled that GRHL factors play crucial roles in establishing the EMT spectrum and moderating the EMT/MET dynamics during cancer progression. Importantly, comprehending the biology of such intermediate or hybrid trans-differentiation states is essential to combat clinically challenging issues such as metastatic aggressiveness and therapeutic resistance (Santamaria et al., 2017; Jolly et al., 2018, 2019; Williams et al., 2019).

### GRHL Family in Other Human Diseases

Craniofacial development encompasses the patterning of bones, muscles and vasculatures of face, skull and jaws, governed by highly coordinated migration signals and spatiotemporal regulation of genetic and molecular factors. Owing to the gene nomenclature, *Drosophila* larvae carrying *grh* mutations have pronounced deformation in the chitinous head-skeleton morphology generating a granular head appearance (Bray and Kafatos, 1991). Similarly, deregulation of Grhl/GRHL factors are also heavily associated in the etiology of craniofacial malformations in mammals (Carpinelli et al., 2017). The failure of cranial neural tube closure in these mutants resulted in anterior spina bifida, prematurely apposed skull bones, split-face, defective neural fold elevation, cranioschisis and exencephaly, lumbosacral spina bifida (open neuropore) and a curled tail phenotype. Accordingly, multiple independent reports have observed that patients with craniofacial malformations are associated with microdeletions of a gene cluster at the chromosomal region 8q22.2-q22.3, comprising clinically relevant genes including *GRHL2* (Kuechler et al., 2011; Kuroda et al., 2014; Sinajon et al., 2015; Chen et al., 2017; Hoebel et al., 2017; de Vries et al., 2020). Dominant-negative mutations in *GRHL3* have been reported in the congenital disorder Van Der Woude syndrome, which is characterized by cleft lip and/or cleft palate (Peyrard-Janvid et al., 2014; Wang et al., 2016; Eshete et al., 2018).

Defects in neural tube closure generate severe congenital morbidity and mortality in human, which occurs at a high rate of 1 in every 1000 human pregnancies (Bhandari and Thada, 2020). Murine models carrying *Grhl2* and *Grhl3* conditional deletions are embryonically lethal with severe defects in organogenesis, dorso-lateral hinge point formation during neurulation and neural tube closure (Ting et al., 2003; Rifat et al., 2010; Werth et al., 2010; Pyrgaki et al., 2011; Menke et al., 2015; Goldie et al., 2016). Gene targeting in mice have demonstrated that the lack or surplus of Grhl2/Grhl3 expression could interfere with spinal neural tube closure (Gustavsson et al., 2007; Brouns et al., 2011; Nikolopoulou et al., 2017; De Castro et al., 2018). In particular, Grhl2 mediates the upregulation of cell-cell junction proteins via modulating the local actomyosin-dependent mechanical stress, which is essential for spinal neural tube closure (Nikolopoulou et al., 2019). The regulation of GRHL2 in the transactivation of OVOL1/2, ESRP1/2, miR-200 family and the suppression of ZEB1 expression during MET is also recapitulated during palate closure (Carpinelli et al., 2020). Furthermore, mouse models carrying *Grhl3* dependent gene manipulation in the surface ectoderm showed severe defects in neural tube closure and open spina bifida (Camerer et al., 2010; Kimura-Yoshida et al., 2015; Molè et al., 2020).

Hearing impairment is another pathologic condition linked to *GRHL2* mutation, where sequencing of the gene loci DFNA28 on chromosome 8q22 in a large American family is associated with progressive autosomal dominant hearing loss (Peters et al., 2002). The study initially identified a frameshift mutation 1609-1610insC generating the *GRHL2* transcript with a premature stop codon in exon 14. A decade later, a second novel splice site mutation in *GRHL2* – c.1258-1G > A resulting in p. Gly420Glufs0111 frameshift mutation in exon 10 was associated with age-related, post-lingual hearing loss (Vona et al., 2013). Subsequent studies have reported that mutations and/or gene polymorphisms in *GRHL2* are implicated in hereditary and acquired hearing loss such as age-related hearing impairment, non-syndromic hearing loss, sudden sensorineural hearing loss and noise-induced hearing loss in Chinese, Korean, Roma and Hungarian populations (Kim et al., 2015; Zhang et al., 2015; Lin et al., 2016; Xu et al., 2016; Li X. et al., 2019; Matyas et al., 2019; Wu et al., 2020). Although mutations and gene polymorphisms of *GRHL2* are associated with multiple hearing abnormalities, substantial association of *GRHL2* in the development of the inner ear is not yet demonstrated. However, a recent examination of deafness genes in the non-human primate model marmoset (*Callithrix jacchus*) has revealed that GRHL2 expression is prevalent in cochlear duct lining cells, hair cells, and supporting cells of the inner ear (Hosoya et al., 2016), denoting that the definitive role of GRHL2 in the inner ear development needs further investigations.

## Epigenetic Control of Grainyhead and GRHL Members

On top of gene expression regulations through *cis*- and *trans*-regulatory elements, epigenetic modifications such as methylation of DNA cytosine and extensive post-translational modifications occur on the core octamer histone proteins collectively modulate chromatin landscape of underlying genes, and thereby regulate gene expression. Although methylation of DNA is a global phenomenon, concentrated methylation on short patches of CpG dinucleotide repeats (CpG islands) are often observed in genes that are suppressed at a particular cell state/type (Suzuki and Bird, 2008). A staggering number of histone modifications occur on the flexible N- and C-terminal ‘tail’ domains. These include prominent alterations such as lysine acetylation, lysine/arginine methylation, serine/threonine/tyrosine phosphorylation as well as many under-examined, minor changes such as lysine ubiquitination/sumoylation, citrullination, ADP-ribosylation, and proline isomerization (Bannister and Kouzarides, 2011). Alterations of CpG methylation patterns and histone modifications are observed in a variety of important cellular processes such as during cellular growth, differentiation, and cancer progression (Rothbart and Strahl, 2014; Audia and Campbell, 2016). GRHL factors have been shown to induce changes in CpG methylation levels and histone modifications during development.

In mouse kidney cells, depletion of active histone marks H3K4me3 and H3K9/14ac are observed at the E-cadherin promoter region exclusively in Grhl2-knockdown cells, denoting that Grhl2 expression is essential to sustain activating histone marks at the promoter (Werth et al., 2010). Selective expression of uroplakin II (*UpkII*) in mouse bladder epithelial cells is also regulated through Grhl3-mediated active H3K9ac mark enrichment on the *UpkII* promoter (Yu et al., 2009). During the development of kidney ureteric buds and collecting ductal epithelia, GRHL2 strongly associates with the active H3K4me3 mark of target genes (*Cdh1*, *Rab25*, *Ovol2*, and *Cldn4*) that are essential for lumen expansion and barrier formation (Aue et al., 2015). Besides direct transcription controls, GRHL2 imposes several epigenetic modifications on selected epidermal differentiation genes. GRHL2 overexpression in normal human epidermal keratinocytes leads to the inhibition of methylation at the CpG island of the *hTERT* promoter and restores hTERT expression, potentially by hindering the activity of DNA methyltransferase DNMT1 (Chen et al., 2010). In addition, GRHL2 overexpression in normal human epidermal keratinocytes also leads to the enrichment of H3K27me3 repressive mark, while simultaneously inhibiting the recruitment of histone demethylase Jmjd3 to the cognate promoters of epidermal differentiation genes such as involucrin (*IVL*), keratin 1 (*KRT1*), filaggrin (*FLG*) and cyclin dependent kinase inhibitor 2A (*INK4A*) (Chen et al., 2012). During epidermal differentiation, GRHL3 binds directly to the *TGM1* promoter to control the expression of transglutaminase (*TGM1*) (Hopkin et al., 2012), a Ca^2+^-dependent enzyme is essential for the formation of cornified cell envelope (Eckert et al., 2005). GRHL3 further recruits the Trithorax complex components MLL2 and WDR5 to the target promoter and increases the active H3K4 methylation mark and drives *TGM1* expression during epidermal differentiation (Hopkin et al., 2012). These studies substantiate the notion that GRHL factors have the potential to epigenetically modify chromatin states during cellular differentiation.

## Pioneer Activity of Grainyhead and GRHL Family

About two meters long, the double stranded DNA is condensed and packed into a typical eukaryotic interphase nucleus that only measures about six micrometers in diameter. This composite level of condensation starts primarily by wrapping the DNA around an octameric protein complex made of four core histones into a structure called nucleosome. Arrays of nucleosomes undergo further condensation into higher-order chromatin structures that functionally demarcate the chromatin boundaries into densely condensed heterochromatin and relatively less compressed euchromatin units. Although the high level of DNA packaging significantly deals with containing the genetic material in a miniscule space, DNA access to gene regulatory proteins during key cellular process such as transcription is greatly restricted. In this context, one crucial question is “How do TFs find their way to gene regulatory elements that are repressed or latent amidst these convoluted nucleosomal barriers in order to initiate transcription for diverse cellular processes?” A new set of regulatory proteins called ‘pioneer factors’ have been identified to accomplish this phenomenal task (Zaret and Carroll, 2011).

Pioneer factors belong to a unique class of TFs that can recognize and bind specific *cis*-regulatory units within permissive heterochromatin, and subsequently prime the chromatin for additional factors to bind, prior to transcription initiation (Zaret and Mango, 2016; Mayran and Drouin, 2018). The pioneer factors display few salient characteristics that are usually lacking in general TFs: (i) the ability to destabilize chromatin compaction (nucleosomes) and to bind to otherwise inaccessible heterochromatin regions, in a cell type/state-specific manner; (ii) the potential to alter pre-existing epigenetics modifications (such as DNA methylation and histone modification) to enhance DNA accessibility; and (iii) the remodeling of adjacent chromatin landscape to facilitate the binding of non-pioneer TFs prior to transcription initiation. In mammals, pioneer factors such as FOXA1, FOXD3, GATA-3, and PU.1 have crucial roles in development, cell fate conversions and deregulation of such factors in also implicated in cancer (Magnani et al., 2011; Iwafuchi-Doi and Zaret, 2016). Compelling evidences have accumulated to illustrate the novel role of Grainyhead and GRHL family proteins as pioneer factors in multiple cellular contexts.

### Pioneering Role of Grainyhead and GRHL in Remodeling Target Enhancers

Pioneer factors can remodel the chromatin landscape to expose functional *cis*-regulatory elements (such as enhancers) that recruit the binding of transcription factors, cofactors and collectively form a stable regulatory complex. Promoters are usually a minimal stretch of DNA sequences, located in proximity to the transcription start sites within a nucleosome-free chromatin landscape to enable easy access to the transcription machinery. In contrast, enhancers tend to be located far (either upstream or downstream) from the cognate promoters and enhance transcriptional outputs in a cell-type/state specific and spatiotemporal manner (Wittkopp and Kalay, 2012; Long et al., 2016). As a pioneer factor, Grainyhead and GRHL proteins bind to enhancers and regulate chromatin accessibility at the target genes during several developmental processes. During *Drosophila* eye development, the unbiased genome-wide characterization of direct TF interactions with enhancer regions of target genes have revealed a significant enrichment of Grainyhead in large fraction of active enhancer regions, which further elucidates the abundant Grh expression in the eye disc (Potier et al., 2014). The utilization of high-throughput genome-wide association methods such as the single-cell assay for transposable-accessible chromatin using sequencing (scATAC-seq) and quantitative trait loci for chromatin accessibility (caQTL) across a panel of *Drosophila* strains have revealed that the pioneer binding of Grh is essential for the opening and accessibility of epithelial cell enhancers (Jacobs et al., 2018). In these Grh binding sites, about 75% of Grh target sites are inaccessible due to the lack of *Grh* expression in the non-epithelial larval brain, whereas the ectopic overexpression of *grh* in the larval brain tissue profoundly increases the chromatin accessibility of these regions (Jacobs et al., 2018). These findings reiterate that, in *Drosophila*, Grh is the chief pioneer factor of the epithelial chromatin landscape. It potentially binds to the recognition sites and alters the closed chromatin landscape of non-epithelial tissues. In addition, the pioneering activity of Grh is also subjected to spatio-temporal and tissue specific regulation. Its pioneering activity has been reported to become essential during and after gastrulation but not during early embryogenesis (Nevil et al., 2020).

**FIGURE 2:**
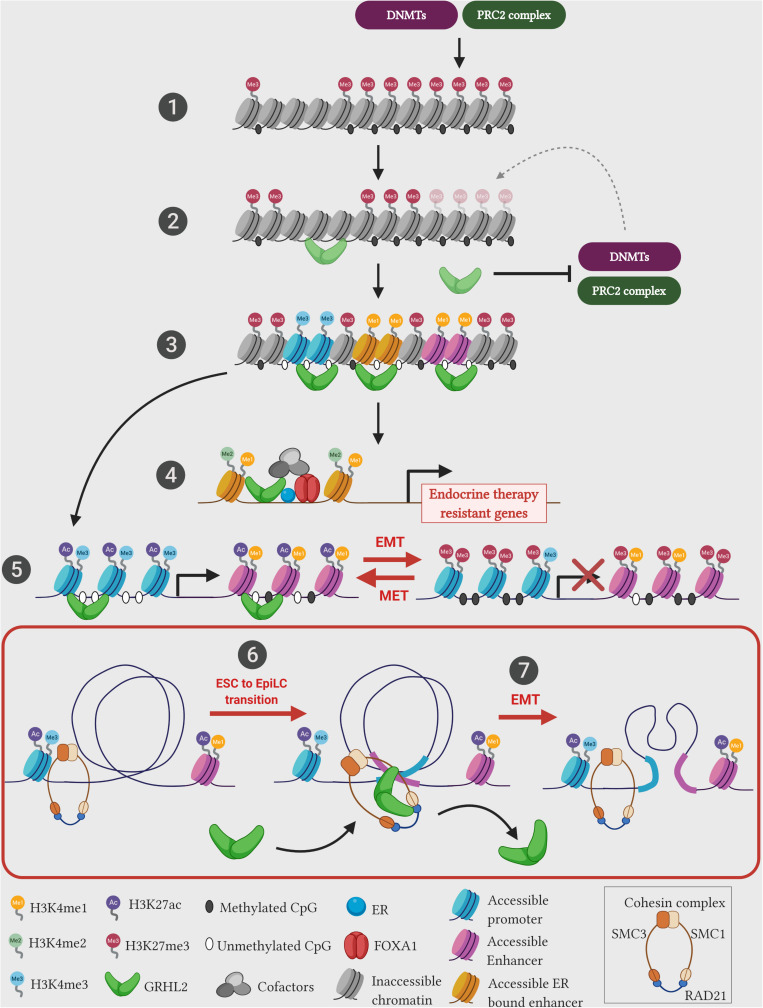
Emerging pioneering functions of GRHL2 during pathophysiology. Recent studies unearth the novel function of GRHL2 as a leading pioneer factor. (1) Typical chromatin landscape comprises cellular DNA wrapped around the core histone complex to form a nucleosome. Over half of the genome remains either in a latent/poised state with no histone marks/methylation status or in a repressed state studded with repressive chromatin marks (H3K27me3)/methylated CpG sites. These epigenetic marks are mediated through epigenetic repressors such as polycomb repressive complex 2 (PRC2) complex, and DNA methyltransferases (DNMTs). (2) Pioneer factors such as GRHL2 can potentially bind and activate latent chromatin in the context of ESC to EpiLC transition (6), or inhibit the activities of epigenetic repressors in the context of EMT (5). (3) GRHL2 primed regions generate accessible chromatin with unmethylated CpG islands and permissive histone marks (H3K4me3 – promoter; H3K4me1 – enhancer). (4) GRHL2 cooperates with pioneer factor FOXA1 at ER bound-active enhancer regions (studded with active histone H3K4me1/me2 marks) to drive transcription of endocrine therapy resistant genes. (5) In the presence of GRHL2, epigenetic landscape of epithelial genes are modified into a permissive chromatin (studded with H3K27ac and H3K4me3) at promoter and enhancer regions, whereas during cellular differentiation such as EMT, lack of GRHL2 expression results in change of chromatin setting of epithelial genes to a repressed state (hypermethylated promoters and enrichment of repressive H3K27me3 mark). (6) During ESC to EpiLC transition, GRHL2 associates with the cohesin component SMC1 on EpiLC-specific enhancer sites to facilitate transition. (7) In epithelial ovarian cancer cells, GRHL2 associates with the cohesin component RAD21 and brings distantly located gene regulatory elements in close proximity, to drive the expression of early epithelial genes such as *ERBB3* and *PERP*. However, SNAI1-mediated EMT induction in these cells downregulates GRHL2 expression potentially disassembles cohesin structure, leading to reduced epithelial gene expression. Illustration created with Biorender.com.

Investigations in mammalian GRHL factors also showed such pioneering roles in modulating the chromatin landscape of enhancers. During the early transition from the mouse naive embryonic stem cells (ESCs) to the primed pluripotent epiblast-like cells (EpiLCs), GRHL2 binds to latent enhancers (regions with low or no histone marks) and restores the epigenetic landscape toward an activation state (high levels of H3K4me1, H3K27ac histone marks and depletion of nucleosomes) of key genes that control the epithelial state and cell adhesion (Chen et al., 2018a; Figure 2). Importantly, in the ESC state, a different set of enhancers and TFs (such as KLF4, KLF5, and EKLF) controls the expression of the above mentioned genes, whereas in the EpiLCs, the control of gene expression switches toward the GRHL2-bound enhancers. Assaying across 47 human cell types, the positional distribution of TF binding motifs within the nucleosome-depleted enhancer sites have shown that GRHL1 is one of the six transcription factors that modulate DNA accessibility (Grossman et al., 2018). This study further elucidates that GRHL1 stably binds to the DNA with prolonged occupancy denoting that it may act in generating central anchor regions for potential transcription initiation. These results posit that in addition to pioneering the chromatin architecture, GRHL factors potentially mediate a major enhancer-switching phenomenon during cellular differentiation.

GRHL proteins also exhibit the role of pioneering the enhancer landscape in human cancers. Chromatin states that denote the accessibility of the genomic region (such as active, repressed, heterochromatin, bivalent and poised) could be annotated using an automated ChromHMM algorithm (Ernst and Kellis, 2012). In ovarian cancer, using experimental ChIP-seq data derived from five major histone marks (H3K4me3, H3K4me1, H3K9me3, H3K27me3, and H3K27ac), our group has utilized this pipeline to show that upon the loss of GRHL2, a radical shift from an active chromatin state toward a latent, poised/bivalent, or repressed chromatin state occur across intronic and intergenic regions at the GRHL2 binding sites of epithelial genes such as *MARVELD3*, *ESRP1, GRHL1, RAB25, OVOL2* and *MUC20* (Chung et al., 2019). Upon re-expressing GRHL2 by using the inducible system, the chromatin changes of these GRHL2 binding sites located at the promoter and enhancer regions further shine the light on the pioneering function of GRHL2. GRHL2 is highly effective to induce MET in ovarian cancer cells with the intermediate phenotype. This is achieved via the suppression of PRC2 activity and to remove the repressive histone mark (H3K27me3) at the promoters with the corresponding suppression of HDAC activity at the enhancers to restore the H3K27ac mark (Chung et al., 2019). However, the pioneering capacity of GRHL2 might differ depending on cellular states along the EMT spectrum. The MET reversibility of GRHL2 in the highly mesenchymal cells has been quite limited suggesting that there would be state-specific pioneering reprogramming mechanisms. In human breast cancer cells, transient siRNA-mediated knockdown of all three GRHL orthologs show a reduced chromatin accessibility on GRHL-regulated enhancer elements that encode proteins required for cell-cell adhesion such as protocadherin-1 (*PCDH1*) and serine peptidase inhibitor 1 (*SPINT1*) (Jacobs et al., 2018). After priming the chromatin into an accessible regulatory landscape mediated by pioneer factors, tissue specific transcription factors and additional cofactors assemble to carryout gene expression. Such regulation is observed in the specific recruitment of GRHL factors to control steroid hormone-mediated gene expression in hormone-dependent cancers. Estrogen receptor α (ER) is a nuclear hormone receptor that drives over 70% of aggressive breast cancers. GRHL2 expression significantly correlates with ER-positive breast cancer tumors (Carroll et al., 2005; Xiang et al., 2012; Werner et al., 2013). ChIP-seq profiling of ER and phosphorylated ER at S118 (pS118-ER) occupancy sites shows a significant overlap with GRHL2 binding motifs (Helzer et al., 2018; Holding et al., 2019). This indicates that GRHL2 occupancy in the ER binding sites potentially drives ER transcription complex. The ER chromatin interaction and the subsequent gene expression changes are mediated through the pioneering activity of FOXA1 on cognate regulatory sites, independent of estrogen hormone signaling (Hurtado et al., 2011; Glont et al., 2019). Indeed, GRHL2 has been identified as the FOXA1 interaction partner at the ER bound-active enhancer regions demarcated with H3K4me1/me2 marks to promote tumor progression (Jozwik et al., 2016). This cooperation between FOXA1 and GRHL2 in ER driven breast cancer cells also contributes toward the resistance to endocrine therapy via the upregulation of LYPD/AGR2 (a receptor/ligand complex) making it a promising targetable frontier in endocrine therapy-resistant tumors (Cocce et al., 2019; Figure 2). These data clearly indicate the inevitable role of Grainyhead and GRHL proteins in the remodeling of gene regulatory units at the targeted sites.

### Pioneering Role of GRHL Proteins in Altering Chromatin Conformation

As mentioned earlier, folding of the chromatin into three-dimensional structures are not only crucial for packaging DNA but also contribute toward fine-tuning of spatiotemporal gene regulation. For instance, in regulation of gene activity by cell-specific enhancers, distal enhancers are brought into close contact with its cognate promoters via DNA looping. Typically, DNA loops can occur between genomic loci which are tens to hundreds of kilobase pairs apart and are referred to as topological associated domains (TADs). The presence of TADs is evident across many species, ranging from *Drosophila* to mammals, and is a conserved feature of the three-dimensional chromatin architecture (Dixon et al., 2012; Hou et al., 2012; Nora et al., 2012; Sexton et al., 2012). These domains are demarcated by boundaries and often enriched in binding of architectural proteins: (i) sequence-specific CCTC-binding factor (CTCF) and (ii) cohesin protein complex, that serves to constrain the DNA loops within the TADs (Parelho et al., 2008; Stedman et al., 2008; Dixon et al., 2012; Rao et al., 2014). Therefore, TADs provide a structural and functional architecture across the genome, which permit short- and long-range chromatin interactions between regulatory elements within the same TADs (intradomain), while limiting interactions that span across the TAD boundaries (interdomain) to ensure proper gene regulation (Lupiáñez et al., 2015, 2016; Flavahan et al., 2016; Hnisz et al., 2016; Weischenfeldt et al., 2017; Beagan and Phillips-Cremins, 2020). The functional consequence of DNA loops in engaging promoters with its distal enhancers to drive transcriptional output of genes have been studied and reviewed (Sanyal et al., 2012; Long et al., 2016). In the context of the Grh family, a handful of studies indicate that GRHL factors have the potential to modulate chromatin looping structures and eventually affect gene expression. During mouse early embryonic development, intron 2 of the *Cdh1* locus potentially functions as an enhancer element that control epithelium-specific E-cadherin expression (Stemmler et al., 2005). Using chromatin conformation capture-based techniques, it was revealed that the recruitment of Grhl2, Grhl3 and Hnf4α to multiple enhancers within intron 2 of *Cdh1* resulted in functional DNA-loops to the Cdh1 promoter, thereby increasing the expression of the epithelial gene (Werth et al., 2010; Alotaibi et al., 2015). Formation of such DNA loops are essential for activating E-cadherin expression in mouse inner medullary collecting duct cells and thereby to induce epithelial differentiation in non-tumorigenic mouse mammary gland cells.

The formation of DNA loops is mainly mediated by the ring-shaped cohesin complex, which consists of four subunits – SMC1, SMC3, SCC1/RAD21, and SCC3/SA1/SA2 that topologically clasps chromatin into looping structures. As revealed by single-molecule imaging studies, the cohesin complex binds to DNA in a ring-shaped conformation and translocate along the chromatin in an ATPase-dependent fashion until it is impeded by CTCF (Davidson et al., 2016; Kanke et al., 2016; Stigler et al., 2016). It has been shown the transient degradation of cohesin resulted in the loss of DNA loops or loop domains, while CTCF degradation lead to the loss of DNA loops at the TAD boundaries, leading to subsequent loss of TAD insulation (Nora et al., 2017; Rao et al., 2017). Therefore, this highlights the role of cohesin complex lies heavily in the formation of loops linking two genomic loci and modulation of cohesin binding affects gene regulation during development and disease (Remeseiro et al., 2013). Recent studies have shown the direct interaction between GRHL factors and members of the cohesin complex. During the transition process of mouse ESCs to EpiLCs, Grhl2 predominantly associates with the cohesin subunit SMC1 to perform its pioneering function at enhancer regions of key epithelial genes to drive the transition process (Chen et al., 2018a). In the investigation on ovarian cancer cell lines, we also show that the co-occupancy of cohesin subunit RAD21 and GRHL2 on the promoter and enhancer elements of early epithelial genes (*ERBB3* and *PERP*) is crucial for their expressions (Sundararajan et al., 2020; Figure 2). Moreover, we show that the recruitment of RAD21 on such enhancer regions are dependent on endogenous GRHL2 expression and the gradual loss of GRHL2 expression along the EMT spectrum of ovarian cancer cell lines might loosen up or alter the chromatin loop structures, which eventually lead to epithelial dedifferentiation. These observations highlight the identification of novel crosstalk between GRHL factors and the chromatin architectural complexes, where the pioneering activity of GRHL factors along the regulatory regions of epithelial genes potentially serve as loop anchors to mediate long-range functional chromatin interactions. Therefore, this mechanism appears to be an essential phenomenon in establishing the epithelial identity in development, while such interactions could be altered during cancer progression under conditions such as EMT. For example, in human sarcoma cells, ZEB1-associated chromatin remodeling factor BRG1 suppresses E-cadherin expression by blocking its promoter region (Somarelli et al., 2016). Depletion of BRG1 in the context of GRHL2 expression further upregulates E-cadherin expression, indicating that ZEB1-mediated chromatin remodeling interferes with GRHL factors associated pioneering function.

## Conclusion and Future Perspectives

Almost three decades ago, foundation studies using *Drosophila* as a model organism have shown that the transcription factor *Grainyhead* is a crucial determinant of the epithelial phenotype and is involved in the development of vital fly organs such as epidermis, trachea, wings, and exoskeleton. Subsequent studies have identified that Grh potentially acts as a transcriptional activator and a repressor to regulate target gene expression, depending on the signaling events and its association with other transcription factors or co-factors. Interestingly, a recent study in the German cockroach (*Blattella germanica*) model has showed that Grh functions as a transcriptional repressor to regulate genes essential for the development of epithelium and molting of old integument (ecdysis) (Zhao et al., 2020). This indicates that the role of Grh in epithelial and epidermal differentiation is fundamental and evolutionarily conserved. Future Grh functional studies on other insect model systems might generate the possibility of Grh-mediated pest control management.

The three mammalian descendants of Grh – Grainyhead like 1-3 are also heavily implicated in the development of vital organs such as the neural tube, epidermis, and craniofacial skeleton. Although mutations and gene polymorphisms in *Grhl* genes were implicated in multiple human abnormalities such as hearing impairment, ectodermal dysplasia syndrome, and cleft palate formation, somatic mutations in *GRHL* genes in human cancer samples occur at a very low frequency (Kotarba et al., 2020). Moreover, the dual functioning of GRHL factors in carcinogenesis and tumor suppression indicate that GRHL factors impose a greater level of control over their target genes and miRNAs. In addition to directly controlling the target gene expression through promoter/enhancer binding, recent studies have shown that GRHL factors are potent modulators of the epigenetic landscape of target genes, which facilitate their spatiotemporal and cell type specific control. Such regulation is prevalent in the maintenance of epithelial barrier functions and the restoration of epithelial phenotypes during EMT/MET fluidity.

Recent studies underline how Grhl factors are essential during surface ectodermal neural lineage specification. Also, members of the GRHL family are potent repressors of the EMT program during development and cancer. These indicate that GRHL factors are at the crossroads of controlling epithelial, mesenchymal and neural-like phenotypes that determine cell lineage and transdifferentiation programs. It is therefore fair to hypothesize that tipping this balance during pathogenesis such as cancer might derail lineage specification, resulting in adverse phenotypes. Therefore, future studies on delineating GRHL factors-mediated of cellular and lineage plasticity through lineage tracing is worth exploring. Such investigations would shed light on the contribution of GRHL factors to the generation of neuroendocrine-like phenotypes, neuroendocrine differentiation observed in several cancers.

Genome-wide research progress in the last decade has brought a novel role of Grh/GRHL members as pioneer factors in limelight. Being pioneer factors, Grh/GRHL2 potentially gain access to the latent and repressed chromatin landscape of epithelial genes and prime such chromatin elements toward transcription initiation. Furthermore, GRHL2/3 associate with protein complexes that control chromatin 3D conformation structures (e.g., cohesins) and potentially regulate their access along the epithelial gene loci during cellular differentiation/dedifferentiation. Of note, chromatin conformational changes during EMT/MET programs during development and cancer progression are starting to emerge recently (Essafi et al., 2011; Yun et al., 2016; Sundararajan et al., 2020). Since GRHL2/3 are recently implicated in modulating the 3D chromatin architecture, future studies employing advanced sequencing techniques like ATAC-seq, FAIRE-seq, and Hi-C or Hi-ChIP would help us comprehend the interplay between GRHL factors and the chromatin accessibility during EMT/MET programs. Such investigations would also clarify our understanding on the dynamic changes of chromatin architecture along the EMT spectrum and eventually pave way toward improved cancer diagnostics and therapeutics.

## Author Contributions

VS and RH conceptualized and wrote the complete first version of the manuscript. All authors contributed to the manuscript revision, read, and approved the final version.

## Conflict of Interest

The authors declare that the research was conducted in the absence of any commercial or financial relationships that could be construed as a potential conflict of interest.
